# Emerging Basic and Clinical Studies on Musculoskeletal Pain and Management

**DOI:** 10.1155/2020/4725303

**Published:** 2020-06-06

**Authors:** Hai-Qiang Wang, Giustino Varrassi

**Affiliations:** ^1^Institute of Integrative Medicine, Shaanxi University of Chinese Medicine, Xixian Avenue, Xixian District, Xi'an 712046, Shaanxi Province, China; ^2^Department of Spine Surgery, General Hospital, Shenzhen University, Shenzhen 518055, China; ^3^Department of Spine Surgery, Longhua District People's Hospital, Shenzhen, China; ^4^Paolo Procacci Foundation, Via Tacito 7, 00193 Roma, Italy

In parallel with life-threatening major diseases such as cardiovascular events, cancers, and diabetes, chronic pain is another leading source of global people's sufferings and disabilities [1]. Pain in the musculoskeletal system seems to be the most common phenotype. More in detail, low back pain is a typical and common disease [2], remaining as top cause of years lived with disability for decades, as revealed by the Global Burden of Disease Study 1990 through 2017 [3]. Patient-reported outcome measures (PROMs) are established indicators reflecting clinical symptoms and pertaining severities. PROMs in musculoskeletal diseases may be studied using the visual analogue scale (VAS), Oswestry disability index (ODI), and EuroQol five dimensions questionnaire (EQ-5D). A linking measure between PROMs and clinical manifestation of patients is the threshold of minimal clinically important difference (MCID) [4]. Other linking modalities detecting pain as phenotypes are novel functional diagnostic imaging [5, 6] and genetic studies identifying underlying genotypes [7]. A number of issues still remain challenging for researchers and physicians, including universal classification schemes [8, 9], diagnostic modalities and criteria [10], and novel treatment strategies for musculoskeletal pain [11, 12]. Accordingly, this special issue seeks to cover musculoskeletal pain-related basic and clinical studies.

In this special issue, readers find eight articles, covering a wide spectrum of musculoskeletal pain. In detail, there are five articles focusing on the management of pain (one on neuromodulation therapy for chronic pain, by R. Staelin et al.; a randomized controlled trial of surgical methods for multilevel lumbar spine stenosis, by S. A. Hamawandi et al.; myofascial physical therapy for chronic pelvic pain syndrome, by K. Grinberg et al.; local ropivacaine pain control for ankle fracture patients, by B. L. Li et al.; and a comparative study for the management of masticatory muscle pain, by B. Saranya et al.). An article by Y. Wang et al. addresses the recovery process for patients with lumbar disc herniation undergoing percutaneous endoscopic lumbar discectomy. One article presents the clinical outcome prediction for adolescents undergoing spinal fusion surgery. One article profoundly analyzes the state-of-the-art of available evidence regarding lateral epicondylitis by K. L. Ma et al. In terms of body regions, there are two articles focusing on extremities (the ankle and elbow; B. L. Li et al. and K. L. Ma et al.), three articles on the spine (S. A. Hamawandi et al., D. D. Ocay et al., and Y. Wang et al.), one on the pelvis (K. Grinberg et al.), and one on the head (B. Saranya et al.). One of the articles is not related to body parts, but describes the potentialities of the neuromodulation therapy (R. Staelin et al.).

Collectively, this special issue presents emerging evidence for musculoskeletal pain in various aspects. In consideration of the high prevalence of pain, it deserves a great attention by the readers.
